# A machine learning based variable selection algorithm for binary classification of perinatal mortality

**DOI:** 10.1371/journal.pone.0315498

**Published:** 2025-01-16

**Authors:** Maryam Sadiq, Ramla Shah

**Affiliations:** Department of Statistics, University of Azad Jammu and Kashmir, Muzaffarabad, Pakistan; King Faisal University, SAUDI ARABIA

## Abstract

The identification of significant predictors with higher model performance is the key objective in classification domain. A machine learning-based variable selection technique termed as CARS-Logistic model is proposed by coupling competitive adaptive re-weighted sampling(CARS) and logistic regression for binary classification. Based on five assessment criteria, the proposed method is found to be more efficient than Forward selection logistic regression model. The CARS-Logistic model is executed to determine the significant factors of perinatal mortality in Pakistan. The identified hazards communicated social, cultural, financial, and health-related characteristics which contain key information about perinatal mortality in Pakistan for policymakers.

## 1 Introduction

By 2030, the fourth Millennium Development Goal (MDG) targeted to reduce neonatal mortality (NNM) to 12 per 1000 live births as it is a key indicator of quality of child health. The Pakistan Demographic and Health survey (PDHS) 2017–18 reported a Perinatal mortality (PM) of 57 per 1,000 pregnancies with an NNM rate of 42 deaths per 1,000 live births. The MDG process evaluation communicated that Pakistan is far from reaching the goal indicating a lack of antenatal facilities and newborn care. The progress in maternal and newborn health in Pakistan during the last decade has produced a slight decline of NNM rate from 55 to 42 death per 1,000 live births, which is insufficient to achieve its under-5 child survival goal. The PM rate ranges from 4 to 7 per thousand births for developed countries and from 20 to 32 for the developing countries [1]. In 2021, an average global NNM rate of 18 per 1,000 live births and 13.9 stillbirths per 1,000 total births were reported, with the highest infant mortality in Sub-Saharan Africa, followed by Central and Southern Asia. Despite significant success in lowering PM over the last thirty years, increased efforts to accelerate progress are still required. The declining trend of PM is much slower than the NNM rate in the world, and it is estimated that 27.8 million children will die by 2030 [2, 3].

The PM rate may be minimized by enhancing and facilitating access to maternity and infant health care services. The most significant determinants of PM in low and middle income countries are pregnancy related infections, intrapartum asphyxia, hypertensive disorder during pregnancy, short birth interval, iron deficiency anemia in pregnancy, congenital anomalies, low birth weight, and Premature birth [4–8]. For Pakistan, short birth interval, youngest and less educated mothers, low income level, and rural area resident are reported as significant variables of high PM [1]. Determination of influential factors of PM using advanced statistical techniques can improve the strategies to achieve the fourth MDG. Selection of important variables has always remained an essential phase of scientific research which originated with modified, efficient and improved techniques in almost every field. Advanced factor selection methods are broadly categorized as supervised and unsupervised learning approaches according to the type of data under study. The logistic regression (LR) model is a widely applicable supervised algorithm as a classifier for categorical data with well-known traditional wrapper-based approaches, including forward selection and backward elimination methods. Adressing measurement error along with factor selection in the context of logistic regression by utilizing corrected responses is recently discussed in literature [9]. A recent python package BOOME is introduced specifically for logistic regression to efficiently handle measurement error in binary responses [10]. The Competitive Adaptive Re-weighted Sampling (CARS) method based on the concept of survival of the fittest coupled with partial least squares regression is presented as a well-structured supervised machine learning (ML) variable selection method. The CARS approach possesses the feature to address various types of data [11].

The motivation of this study was to develop an efficient modeling technique in the context of binary factors by combining the CARS approach with the LR model. The proposed modeling algorithm, namely Competitive Adaptive Re-weighted Sampling with LR (CARS-Logistic) is applied on a real data set of PM collected for Pakistan to identify the most significant factors. The novel contribution to this study included:

(i) development of a more efficient approach compared to traditional variable selection methods by integrating the LR model with CARS technique in the context of categorical response.
(ii) Determination of important predictors for perinatal mortality in Pakistan.

## 2 Materials and methods

The proposed method is a unification of the CARS approach with the LR model. The algorithm initiates with regression modeling and then applies the CARS approach for variable selection. The forward selection-Logistic (FS-logistic) regression is used as the standard model for comparison.

### 2.1 Competitive adaptive re-weighted sampling with logistic regression (CARS-Logistic)

The LR model, the most prominent supervised ML algorithm for classification, is used for prediction and variable selection. The backward elimination and forward selection are the most popular traditional methods integrated with LR for removal of irrelevant and redundant variables and determination of influential factors. The basic assumption of LR are removal of outliers, independence of residual terms, and absence of multicollinearity.

Let *Y* be a binary outcome variable and X is a (*n* × *p*) matrix of predictors, then the logit function is defined as the logarithm of the odds of the positive outcome

$$\begin{array}{c} logit\left(Y\right)=ln\left(\frac{Y}{1-Y}\right)=\alpha +cX \end{array}$$ (1)

where *α* and *c* represent the intercept and regression coefficients respectively. The data matrix X embodies **s** samples in rows and **v** variables in columns. The vector **Y** indicates the binary response variable with order *s* × 1. The Monte Carlo (MC) sampling method is adopted with 50 iterations and 10-fold cross-validation is executed. In every succession of MC sampling, an LR model is created by using the randomly selected subsets with the intention of choosing the variables with a high degree of flexibility regardless of variation. Suppose that ***c*_*i*_** are the regression coefficients for predictor variables. Thus, we have the formula,

$$\begin{array}{c} Y=Xc_{i}+e \end{array}$$ (2)

Where *e* is the error term and ***c*_*i*_** is the v-dimensional vector. The absolute value of the ith element in ***c*_*i*_**, denoted by |*c*_*i*_| (1 ≤ *i* ≤ *v*) reflects the *i*^*th*^ variable contribution to **Y**. Thus, the larger |*c*_*i*_| represents the more important *i*^*th*^ variable. A normalized weight to define the importance of variables is defined as,

$$\begin{array}{c} h_{i}=\frac{|c_{i}|}{\sum |c_{i}|},i=1,2,...,v \end{array}$$ (3)

The threshold is set to be <0.05 to eliminate the non-significant variables. Then the exponentially decreasing function (EDF) is utilized to remove the variables which have relatively small absolute regression coefficients by force. In the *i*^*th*^ sampling run, the ratio of variables to be kept is computed using an EDF defined as,

$$\begin{array}{c} q_{i}=ze^{-ti} \end{array}$$ (4)

Where *z* and *t* are two constants determined by the two conditions:

(i) in the first sampling run, all the *v* variables are taken for modeling, which means that *r*_1_ = 1,
(ii) in the *N*^*th*^ sampling run, only two variables are reserved such that we have $r_{N}=\frac{2}{v}$.

With these conditions, z and t can be calculated as:

$$\begin{array}{c} z={\left(\frac{v}{2}\right)}^{1/N-1} \end{array}$$ (5)

$$\begin{array}{c} t=[\frac{ln(\frac{v}{2})}{N-1}] \end{array}$$ (6)

Following EDF based variable elimination, adaptive re-weighted sampling (ARS) is employed to further eliminate the variables competitively to obtain the optimal subset based on the rule of survival of the fittest. The selected variables are subjected to random weighted sampling experiments with replacement possessing dominant weights in each iteration. A 10-fold cross-validation process is applied to assess the model performance during the selection of subsets of the data.

Finally, five model assessment criteria, including Akaike information criterion (AIC), Bayesian information criterion (BIC), McFadden’s Pseudo R squared ($R_{M}^{2}$), McFadden’s Adjusted Pseudo R squared ($R_{adjM}^{2}$), and Cox & Snell Pseudo R squared ($R_{C\&S}^{2}$) are used to test the performance of the standard and proposed methods.

**Fig 1:**
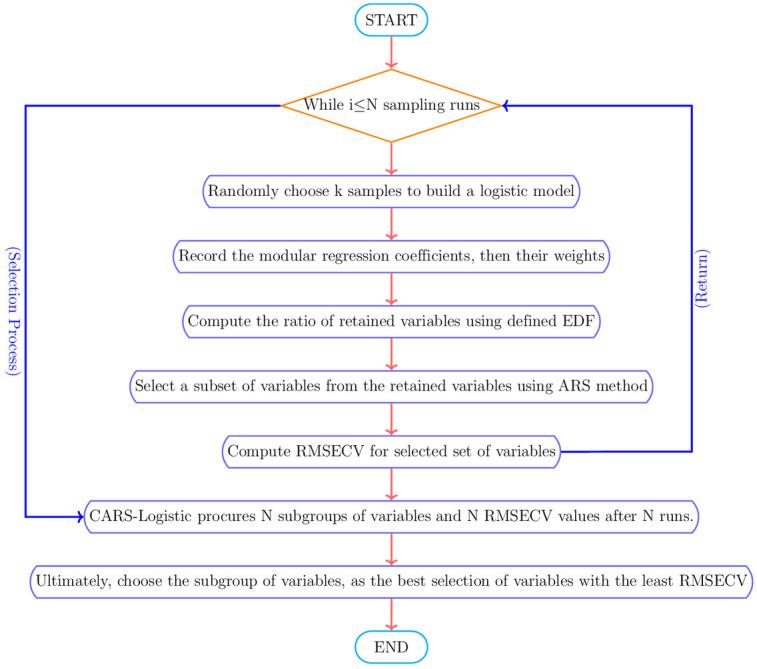
The scheme of CARS-Logistic algorithm.

The general description of the CARS-Logistic algorithm is outlined in Fig 1.

**Fig 2:**
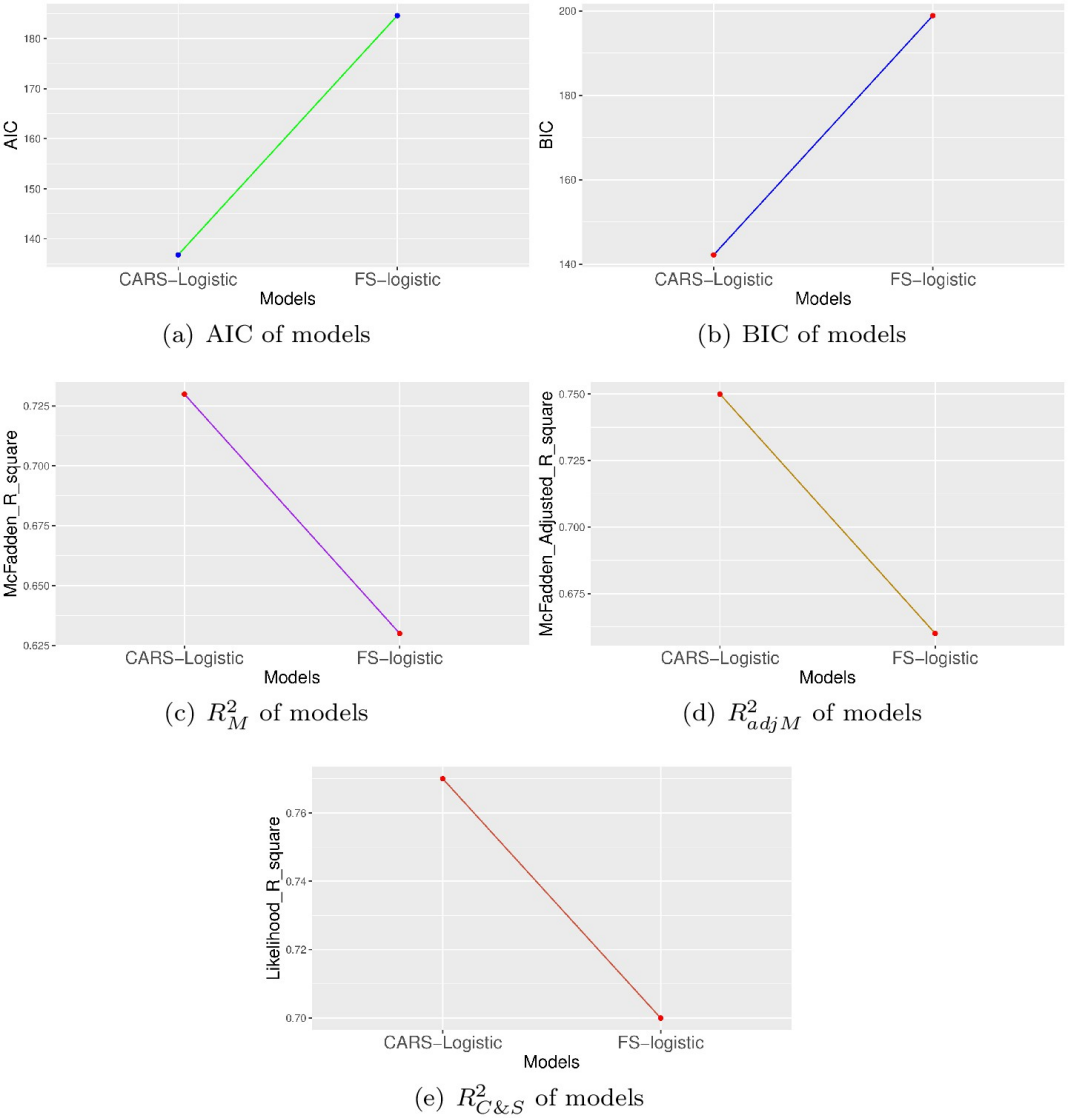
Efficiency comparison of FS-logistic and CARS-Logistic variable selection methods based on five model assessment criteria for simulated data.

Simulated data along with real data set is employed to validate the efficiency of models. The efficiency of FS-logistic model and CARS-logistic approach is compared over simulated data which is purely generated on binary categorical properties. Then performance of both approaches is examined over real data set to further authentication of models Fig 2.

### 2.2 Data simulation

The binomial probability distribution is used to generate simulated data set consisting of 5000 observations and 100 categorical predictors belonging to the binomial family. The data set is generated within the statistical software R.

### 2.3 Real dataset

The data is obtained from the “Pakistan Demographic and Health Survey (PDHS)” 2017-18, which was coordinated by the “National Institute of Population Studies (NIPS)” under the auspices of the “Ministry of National Health Services”, and was facilitated and carried out by (NIPS), Pakistan.

The survey provides data on pregnancy during the five years preceding the survey, along with other information. In this study, perinatal mortality included stillbirths at or after 28^th^ gestational weeks up to deaths of neonates within seven days [12]. A sample of 5436 observations (perinatal births) with 94 predictors having complete data is extracted from primary data. In light of previous literature, 94 indicators regarding birth, demographic, financial, and health are considered for analysis. The occurrence or non-occurrence of perinatal mortality among women of childbearing age is considered as a response variable (**Y**) which is measured as a dichotomous variable with possible values ‘1’ for a woman who has experienced PM and ‘0’ for a woman having a live birth. All the selected predictors are categorized as reflecting normal groups according to the literature and represented by the matrix $X_{i}$ where (*i* = 1, 2, …, 94).

## 3 Results

The CARS-Logistic model is executed on simulated as well as real data set of perinatal mortality. The analysis showed the higher efficiency of proposed algorithmic approach compared to standard logistic modeling technique. Further, the proposed method is executed to select significant factors related to perinatal mortality in Pakistan.

### 3.1 Simulation based results

Using a Binomial distribution, the simulated data set having a binary response and 100 explanantory variables with 5000 samples is generated. The constructed data is then split into test and training sets with the ratio of 70:30 to evaluate the performance of FS-logistic and CARS-Logistic models. The FS-logistic and CARS-Logistic method selected 40 variables and 55 significant predictors respectively for the simulated data set. The results based on five model assessment criteria are represented in Fig 1. The visual display evidenced the higher performance of the CARS-Logistic algorithm compared to the FS-logistic model in terms of accuracy and efficiency.

### 3.2 Perinatal mortality data set

The perinatal mortality data set from the PDHS 2017-18 for initial analysis included a binary outcome of interest, which is an occurrence or non-occurrence of perinatal mortality, along with 94 predictors and 5436 samples.

### 3.3 Assumptions of logistic regression

**Fig 3:**
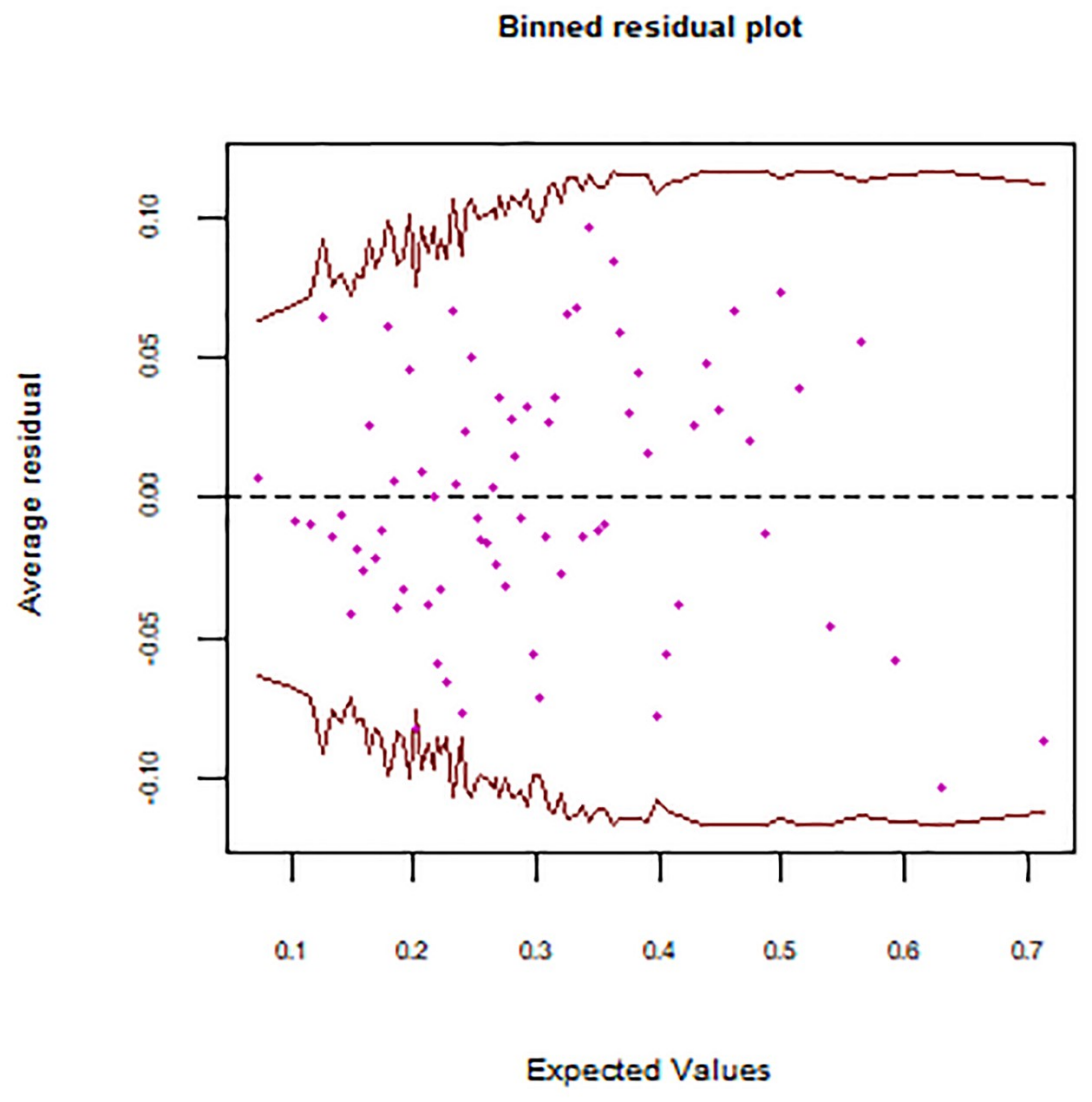
Binned residual plot.

The logistic model is based on the basic assumptions of removal of outliers, independence of residual terms, and absence of multicollinearity. After removing outliers and near-zero variance predictors, 94 explanatory variables are selected for analysis. To check the independence of residuals initially, a binned residual plot is used to avoid the discrete pattern and is displayed in Fig 3.

The pattern of residuals in Fig 3 showed the independence of error terms as the 90% confidence band contained data. Additionally, the residual points depicted the random and arbitrary functioning of error terms, and hence Fig 3 showed that the residuals are independently and randomly distributed.

**Fig 4:**
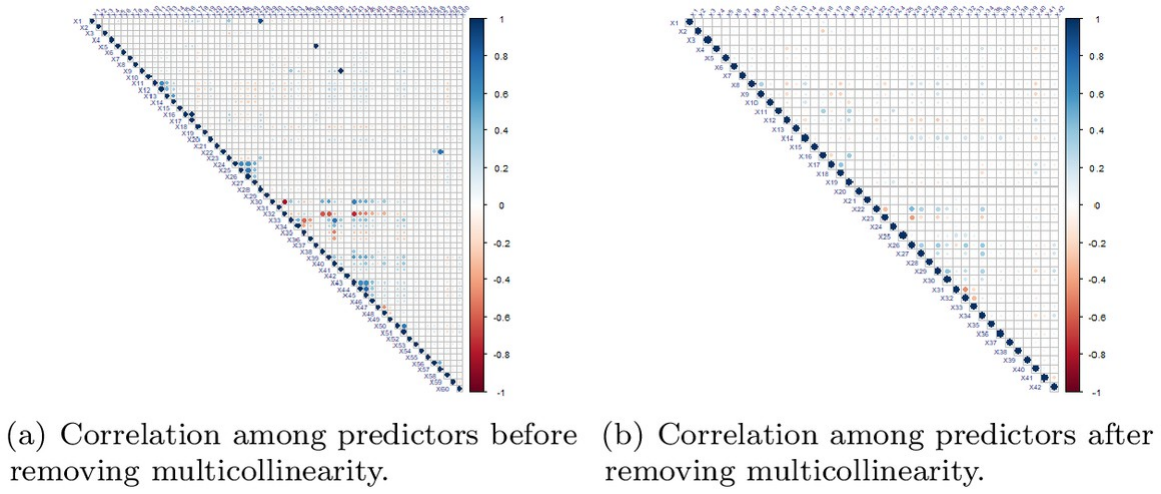
Correlation plots.

To examine the assumption of multicollinearity, the correlation map between predictors is presented in Fig 4a showing positive and negative associations with red and blue dots respectively. The size and intensity of the dots reflected the magnitude and strength of association between predictors respectively. Multicollinearity is identified among 14 explanatory variables having high correlation (> 0.7). Fig 4b presented the correlation map after removing highly correlated predictors to fulfill the assumption. After removing highly correlated variables, 80 predictors are selected for application and comparison of traditional and proposed modeling schemes.

**Fig 5:**
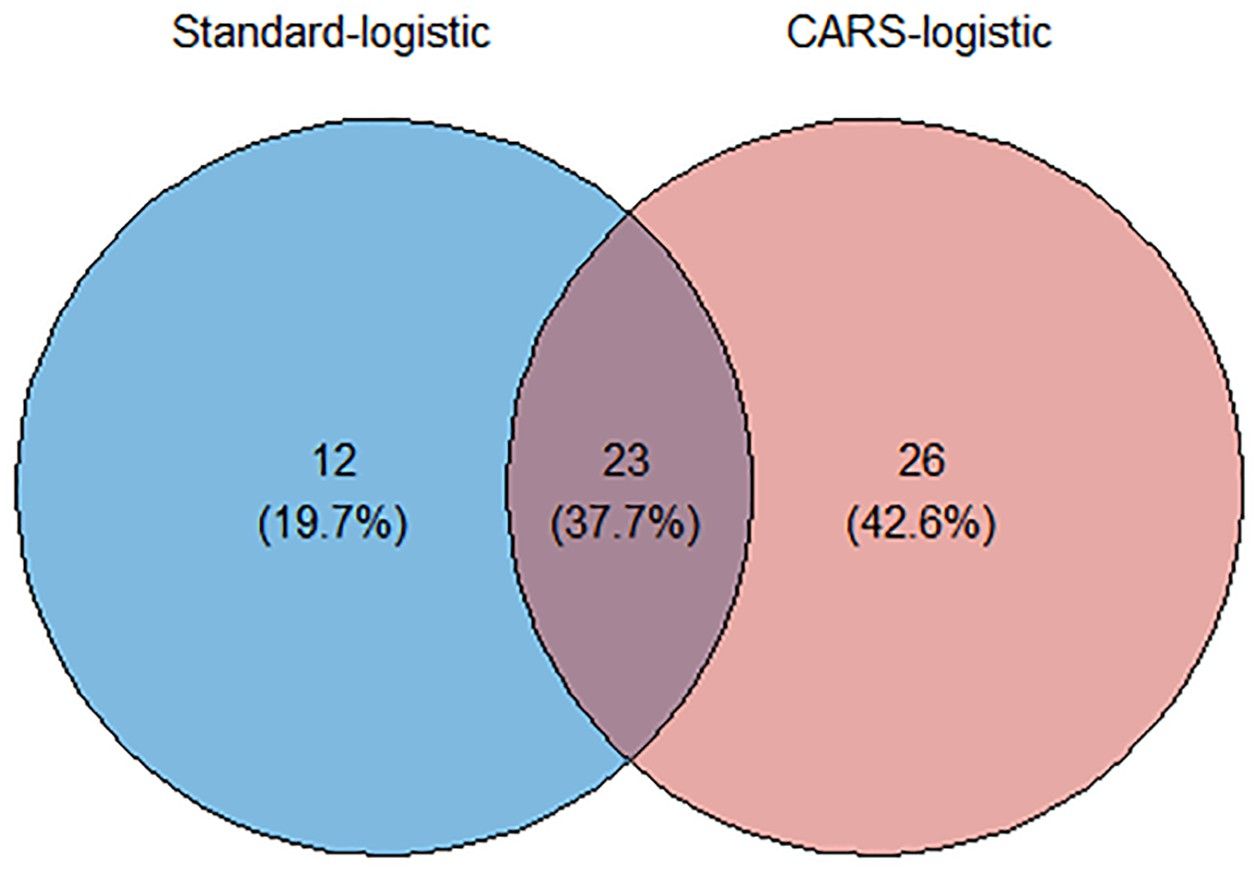
Percentage differences in factors selected by FS-Logistic and CARS-Logistic model.

**Fig 6:**
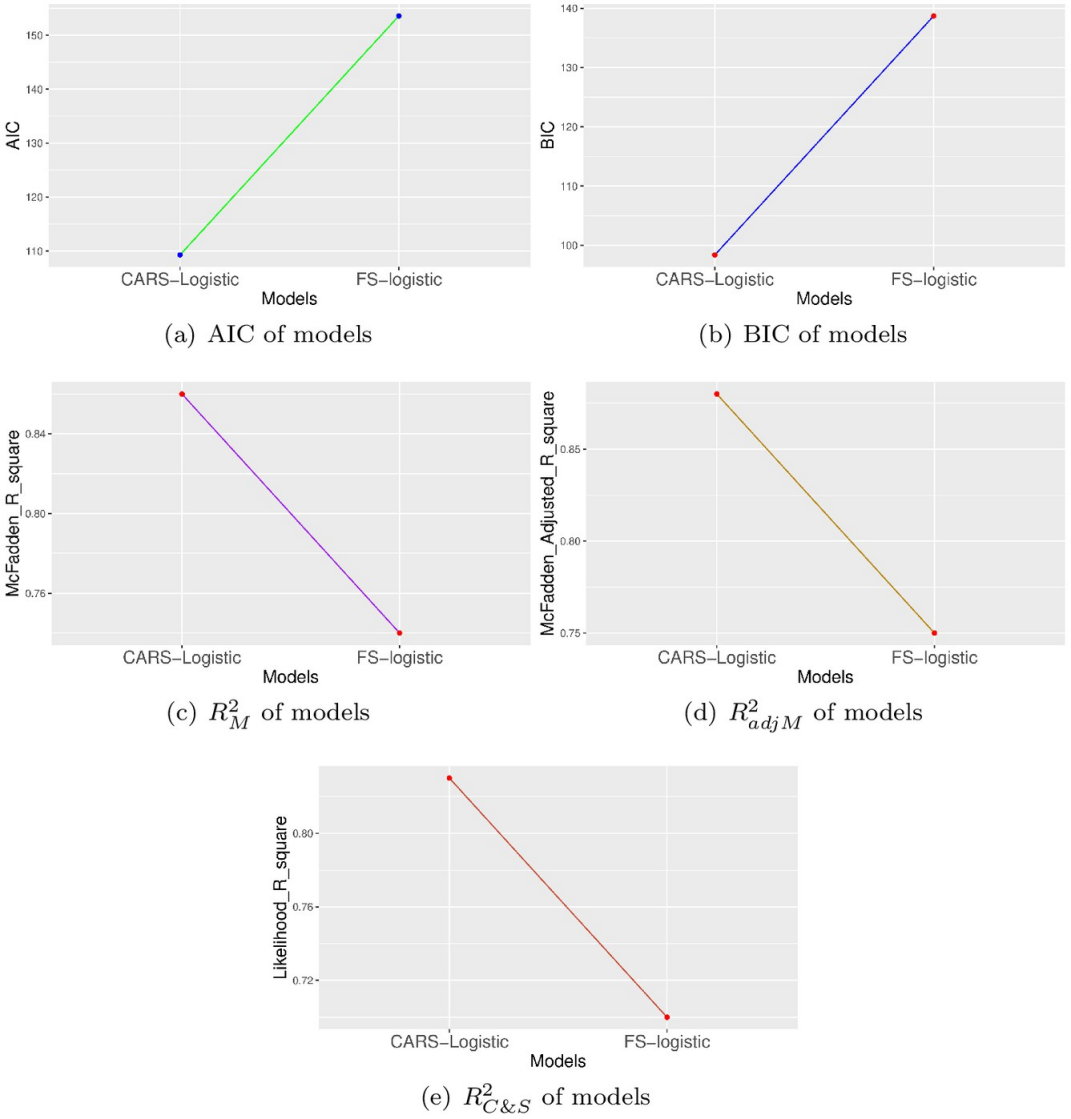
Efficiency comparison of FS-logistic and CARS-Logistic variable selection methods based on five model assessment criteria for real data set of PM.

The final analysis showed that the CARS-Logistic model identified 45 significant variables out of 80 predictors and the FS-Logistic approach established a model comprised of 35 factors. The difference between selection of factors is presented by Venn diagram in Fig 5 displaying 23% common factors selected by both methods. The performance behavior of models presented graphically in Fig 6 showing the lower values of AIC and BIC for CARS-Logistic compared to the Standard-Logistic method and higher values of $R_{M}^{2}$, $R_{adjM}^{2}$, $R_{C\&S}^{2}$ for proposed method compared to traditional technique suggesting the CARS-Logistic algorithm as a better-fitted model in terms of efficiency and variable selection. Table 1 presented the significant factors of perinatal mortality in Pakistan identified by the CARS-Logistic model. From the set of 80 variables, CARS-Logistic selected 45 variables of remarkable importance.

**Table 1:** Regression estimates of the CARS-Logistic alogrithm to identify significant predictors of perinatal mortality.

Factor	Reference group	Odds ratios
Awareness about family planning	Yes	2.41
Marriage to first birth interval (years)	1-3	1.59
Sons died	None	1.45
Daughters died	None	1.33
Worked after marriage	No	1.33
Wanted pregnancy when become pregnant	Yes	1.26
Mother’s age	15-24 years	1.24
Husband’s age	15-25 years	1.24
Getting permission to go for medical help	Not a big problem	1.22
Getting money needed for medical help	Not a big problem	1.20
Literacy	Cannot read at all	1.19
Worked before marriage	No	1.17
Beating justified if wife neglects in-laws	No	1.15
Size of child	Larger than average	1.14
Watching television	No	1.13
Number of antenatal visits	More than 6 visits	1.13
Blood tests taken during pregnancy	Yes	1.21
Sons alive	None	1.12
Had a say in choosing husband	Yes	1.12
Daughters alive	None	1.10
Husband’s education level	Literate	1.09
Medical assistance: traditional birth attendant	No	1.09
Desire for more children	Yes	1.08
Use cigarettes and tobacco	Yes	1.08
Avoid getting Hepatitis B or C	Yes	1.08
Antenatal care: government hospital	Yes	1.08
Antenatal care: private hospital	Yes	1.06
Region of residence	Urban	0.95
Person who usually decides what to do with		
money husband earns	Respondent alone	0.95
Time to get to water source	On premises	0.95
Prenatal check up by nurse/lady health visitor	No	0.95
Relationship to household head	Head	0.93
Age of household head	Less than 30 years	0.92
Blood pressure observed during pregnancy	Yes	0.92
Wealth index	Rich	0.91
Taking iron pills, sprinkles or syrup		
during pregnancy	No	0.91
Household has car	No	0.90
Sex of household head	Male	0.90
Awareness about ovulation	No	0.89
Age at first cohabitation	Less than 15 years	0.88
Type of cooking fuel	Organic	0.87
Time of first antenatal visit (months)	No visit	0.87
Number of births in last three years	2	0.82
Bank account of mother	Yes	0.71
Birth order number	1-5	0.70

The results represented in Table 1 depicted that awareness about family planning, the mother’s age and her marriage to first birth interval, sons and daughter died earlier, desired pregnancy, husband’s age, working status of mother are highly associated factors of PM. Among other important variables, birth order number, bank account of mother, literacy of mother, age of household, and relationship to household head, are included for PM.

It is observed that complexities during pregnancy are caused by the type of cooking fuel, age at first pregnancy, and previous birth history along with terminated pregnancies. Husband’s age, knowledge about own health, and family planning, medical assistance provided, timing and numbers of antenatal check-ups, and visits are also significantly associated with perinatal mortality.

Women’s desire for pregnancy, birth order number, size of child at birth, domestic violence, and tough working conditions during pregnancy or before delivery are found to be prominent causes of infant mortality in Pakistan.

These risk factors are specifically associated with the social settings in Pakistan. The most noteworthy risk factors are region, household facilities, relationship to household head, financial independence of women, medical assistance/care provided, health care provider, husband’s age, size of child at birth, and medical check-ups and tests during pregnancy. The results indicate that women’s health, knowledge about health issues, mental health, and living conditions are prime reasons for PMs.

**Fig 7:**
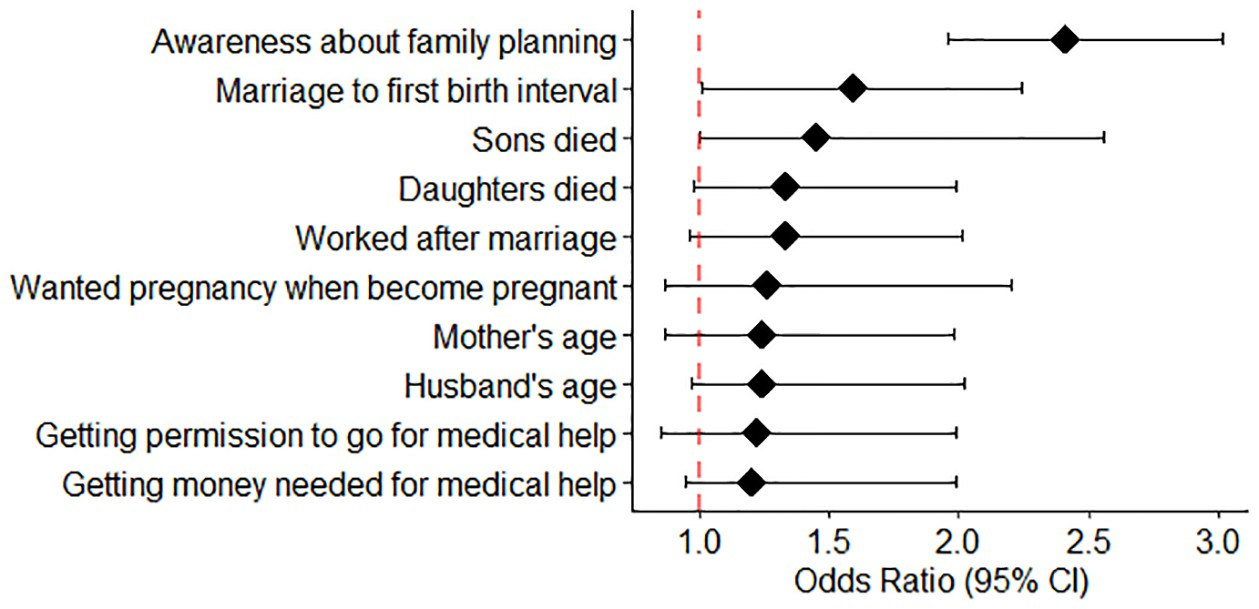
Odds ratio forest plot.

The forest plot is illustrated in Fig 7 representing odds ratio along with 95% confidence interval of 10 most significant variables of perinatal mortality.

## 4 Discussion

The targets of the present study are to propose an efficient ML based variable selection algorithm for binary classification and to identify the significant factors of perinatal mortality in Pakistan. Hence, a recent and advanced ML based factor selection technique is integrated with a logistic regression model to compare with a well-known and widely applicable traditional FS-logistic technique. This study compared two variable selection methods; FS-Logistic method following the forward selection logistic regression model and CARS-Logistic, a method based on the EDF and ARS principles integrated with logistic regression technique. Both subset selection methods are applied to a real data set obtained from PDHS (2017-18) to identify the significant risk factors of PM in Pakistan.

The CARS method was initially proposed in 2009 as a feature selection algorithm for choosing wavelengths. A previous study integrated CARS feature selection method with Partial least squares (CARS-PLS) modeling for regression and compared the proposed CARS-PLS with the Monte Carlo uninformative variable selection method using 10-fold cross-validation. The research proved that the proposed algorithm possess higher efficiency compared to the other method [11]. The current research unified CARS variable selection approach with logistic regression for classification and compared the proposed CARS-Logistic algorithm with a FS-Logistic model using 10-fold cross-validation with interleaved partition type and 50 iterations. Consistent with the literature, the current study also analyzed the functioning of the CARS-Logistic algorithm on simulated and real data sets, observing the higher efficiency of the proposed method over the classical logistic regression model.

Another recent study introduced a novel PLS assisted Excitation-emission matrix fluorescence spectroscopy based analytical procedure coupled with the competitive adaptive re-weighted sampling (CARS) and evidenced that the execution of the proposed method enhanced the efficiency of the calibration models [13]. An additional CARS-successive projections algorithm (CARS-SPA) was introduced as an improved factor selection method to execute multivariate calibration. The authors proved that the proposed approach selected the subset with sufficient information [14]. A study conducted in 2011 demonstrated the higher performance of near-infrared (NIR) spectroscopy integrated with CARS compared to uninformative variable elimination and moving window partial least squares to select the significant wavelengths [15]. Another similar piece of research held in 2016 evaluated the efficiency of CARS with an application of traditional Chinese medicine. The authors reported CARS as a speedy, non-destructive and efficient algorithm in the domain of medicine [15]. An application of the CARS method coupled with random forest for dimension reduction is executed to select significant wavelengths from sorghum spectra is presented in 2023. The study concluded with consistent remarks regarding efficiency and rapidness of the CARS method [16]. Most of the literature supported the final conclusion of the present study with applications of the CARS method separately or in combination with other modeling approaches in a variety of domains.

Finally, the proposed optimal model is used to identify the influential factors of the PM in Pakistan. The social, economical, and health-related factors identified by the present study are consistent with literature for various regions globally [8, 17–19] The current analysis evidenced that the proposed CARS-Logistic method is a more efficient variable selection method than the FS-logistic approach for binary classification. More importantly, this investigation also deduced many important risk factors for perinatal mortality that were previously under-reported or ignored in Pakistan.

## 5 Conclusions

The Proposed CARS-Logistic algorithm is a better choice regarding model performance and variable selection for classification. It indicates that the machine learning-based algorithm generates a classification model with superior interpretation potential. Using the CARS method integrated with a logistic regression model, the risk factors identified as the significant predictors of PM were commensurate with other studies. So, the proposed algorithm has the potential as a more efficient classification technique in public health research.
